# Supplementary material for: Sequential Inflammatory and Matrisome Programs Drive Remodeling of the Mouse Carotid–Jugular Arteriovenous Fistula
Source: Cells. 2025 Dec 16;14(24):1998. doi: 10.3390/cells14241998 (PMC12731578; doi:10.3390/cells14241998)
Supplement: Supplementary file 1 [file cells-14-01998-s001.zip › Supplementary Figures Mouse AVF 120425.pdf]

Figure S1

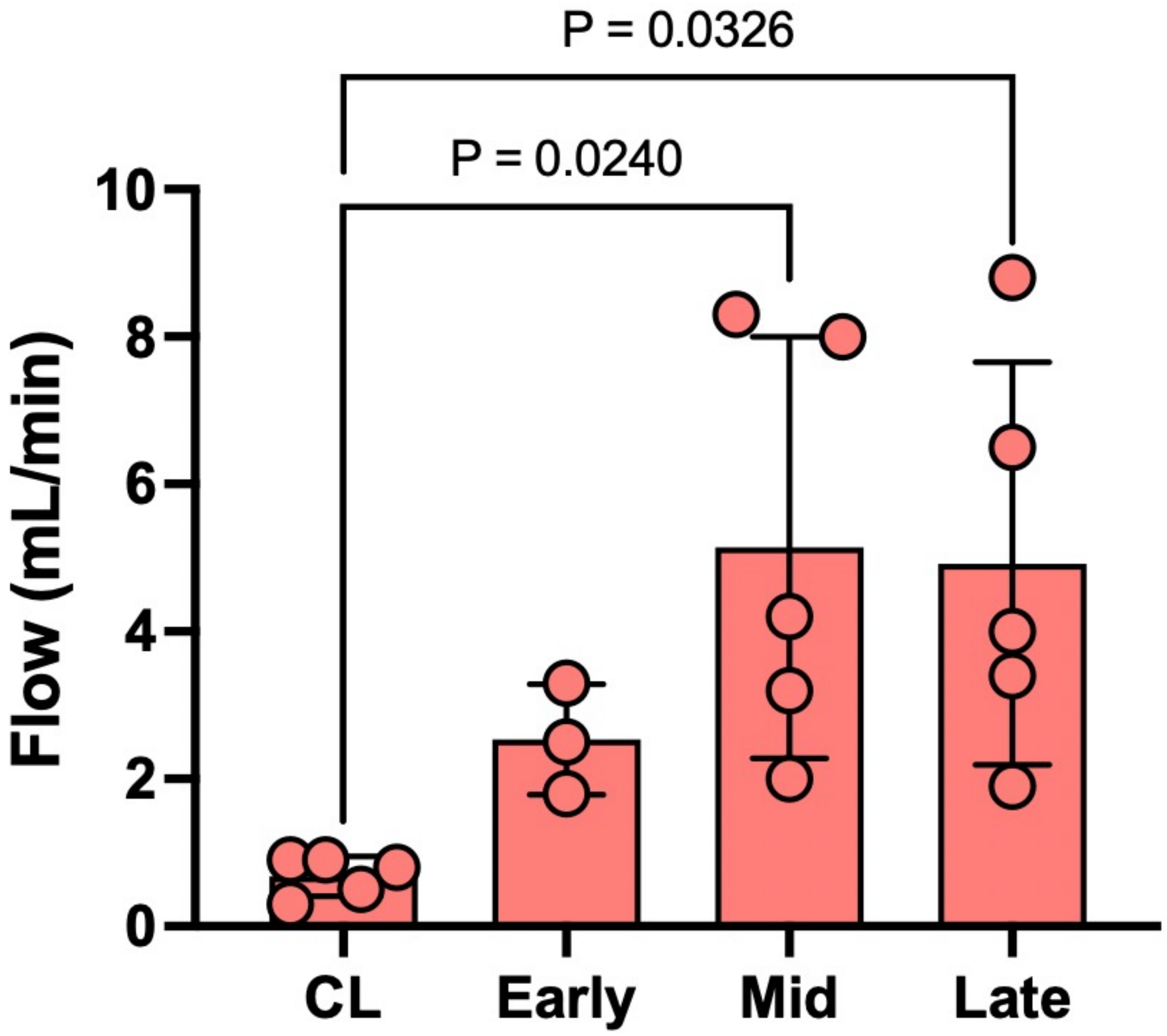

Figure S1. Increase in blood flow after creation of the mouse arteriovenous fistula (AVF). Blood flows of contralateral jugular veins (CL, n=5) and of early (n=3), mid (n=5), and late AVFs (n=5) measured with a Transonic probe before tissue harvest. Groups were compared by ANOVA followed by Tukey's multiple comparisons test. Significant P values are shown.

Figure S2

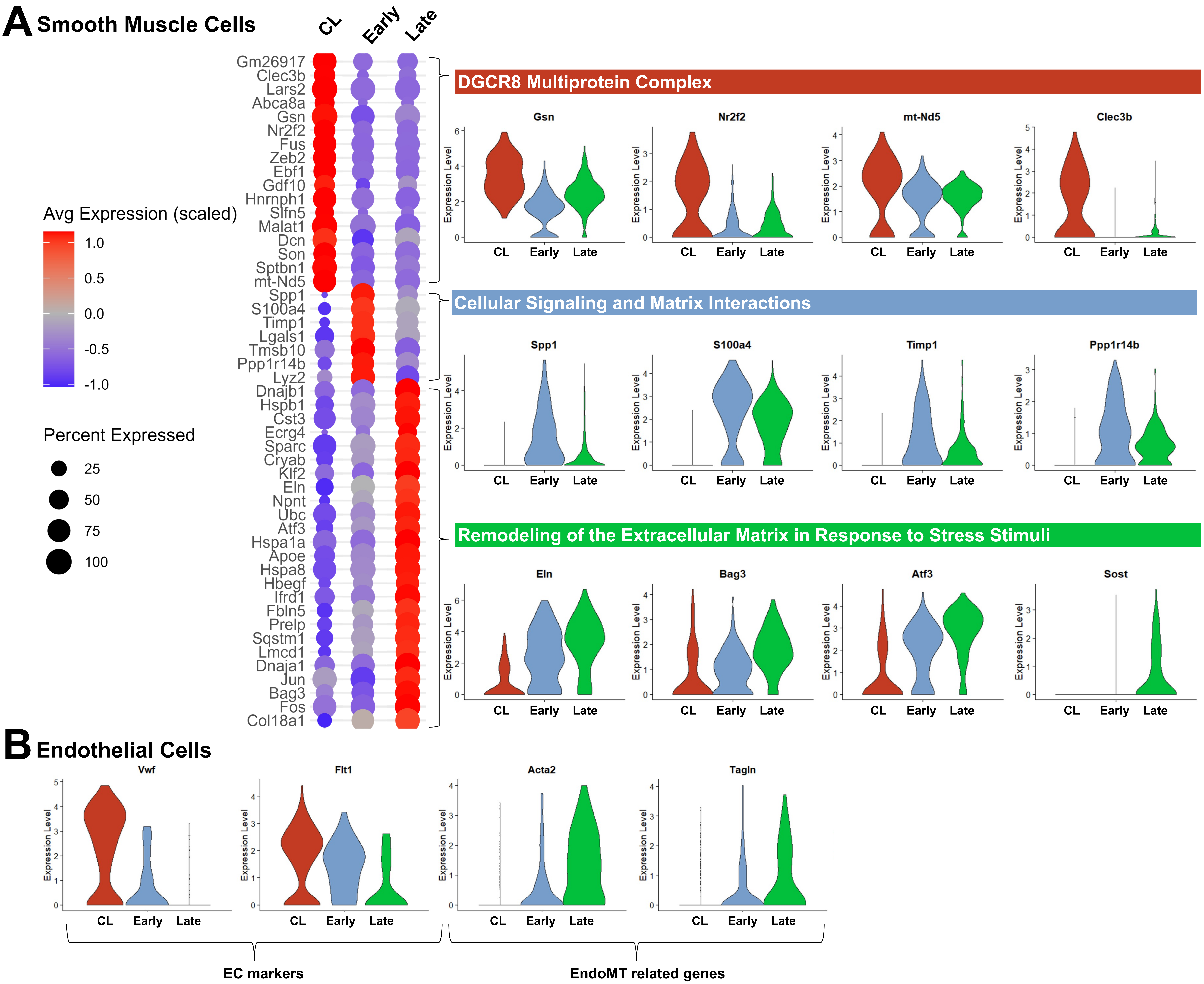

Figure S2. Transcriptional characteristics of smooth muscle cells (SMC) and endothelial cells (EC) after creation of the mouse arteriovenous fistula (AVF). A) Differentially expressed genes among SMCs from contralateral veins (CL) and the outflow veins from early and late AVFs. **B)** Endothelial-to-mesenchymal transformation of venous ECs after creation of the mouse AVF.

Figure S3

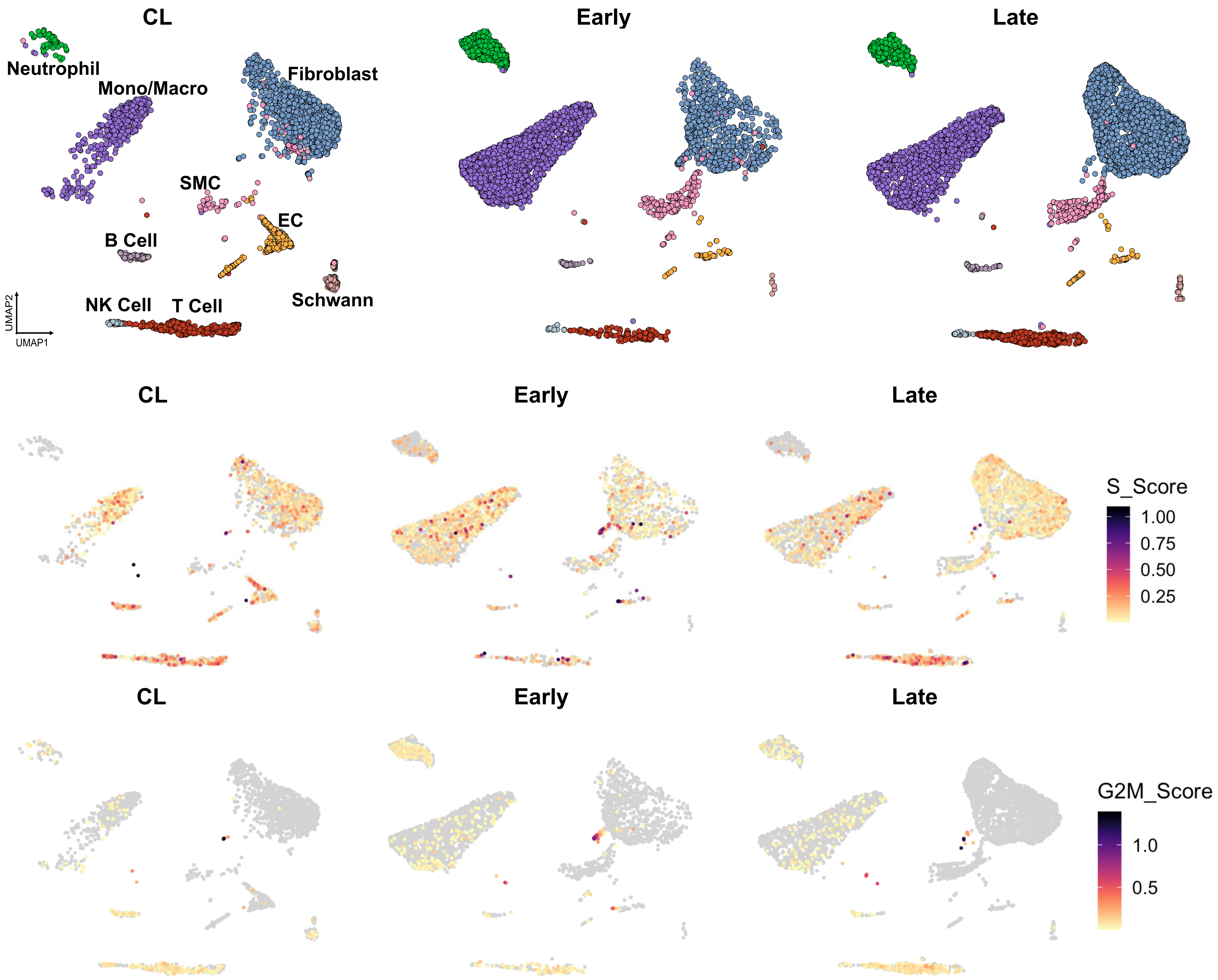

Figure S3. Proliferation scores in cell populations of the mouse arteriovenous fistula (AVF) by scRNA-seq. UMAPs of 10,500 cells from contralateral veins (CL) and the outflow veins from early and late AVFs, indicating the DNA synthesis (S_Score) and cell division (G2M_Score) gene signature scores by experimental group. Scores combine the expression levels of multiple genes with an experimentally curated function.

Figure S4

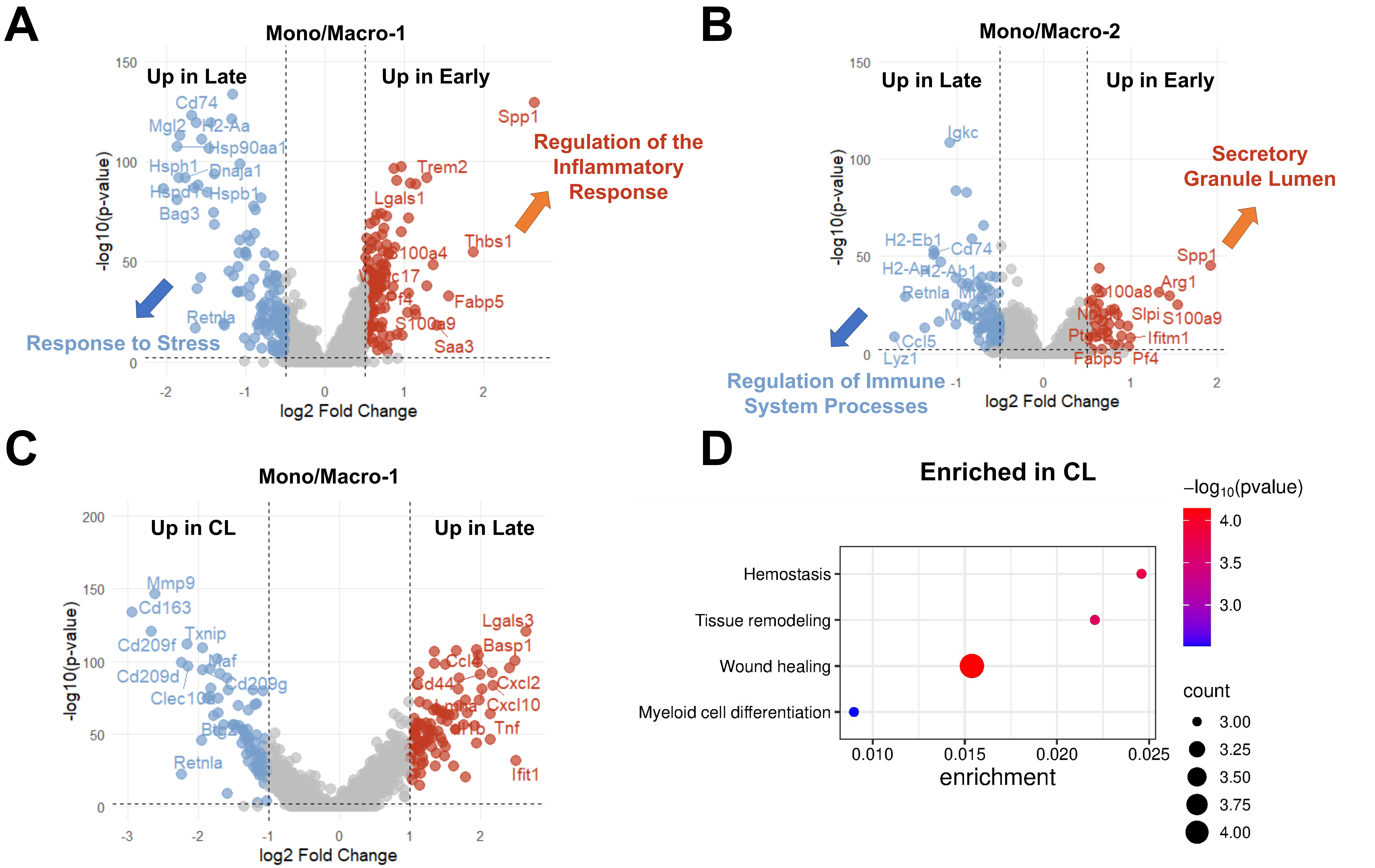

Figure S4. Differentially expressed genes (DEG) in monocyte/macrophage phenotypes after creation of the mouse arteriovenous fistula (AVF). A-C) Volcano plots of DEGs in **(A-B)** mono/macro phenotypes between early and late AVFs, and **(C)** in homeostatic mono/macros between late AVFs and contralateral veins (CL). **D)** Pathways enriched in mono/macros from contralateral veins.

Figure S5

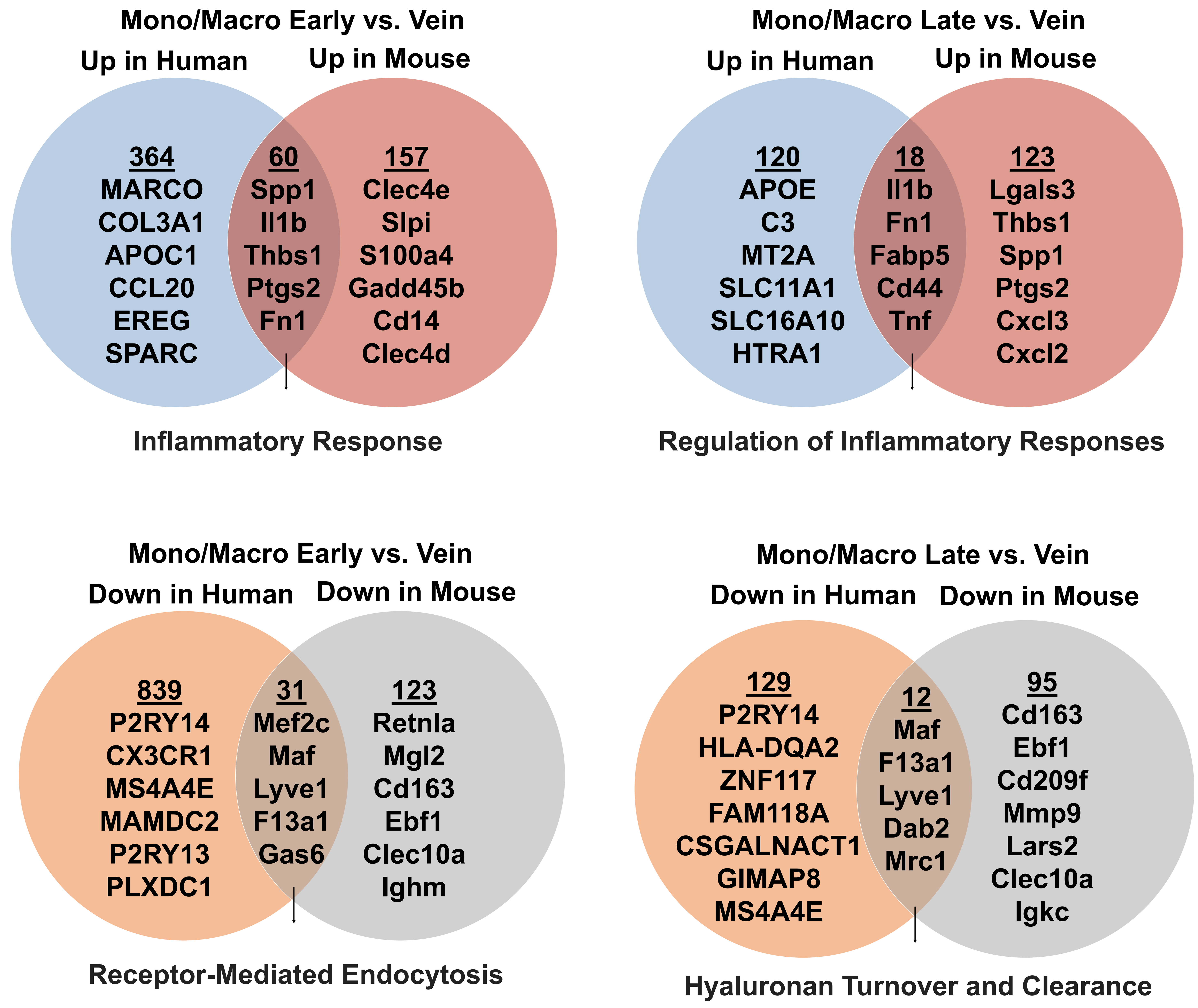

Figure S5. Common differentially expressed genes (DEG) in monocyte/macrophages from mouse and human arteriovenous fistulas (AVF). The top Venn diagrams show upregulated genes in murine early and late AVFs compared with the contralateral veins, and their intersect with upregulated genes in early and late human fistulas compared with pre-access veins. Similarly, the diagrams at the bottom show the cross-species comparative analyses for the downregulated genes.

Figure S6

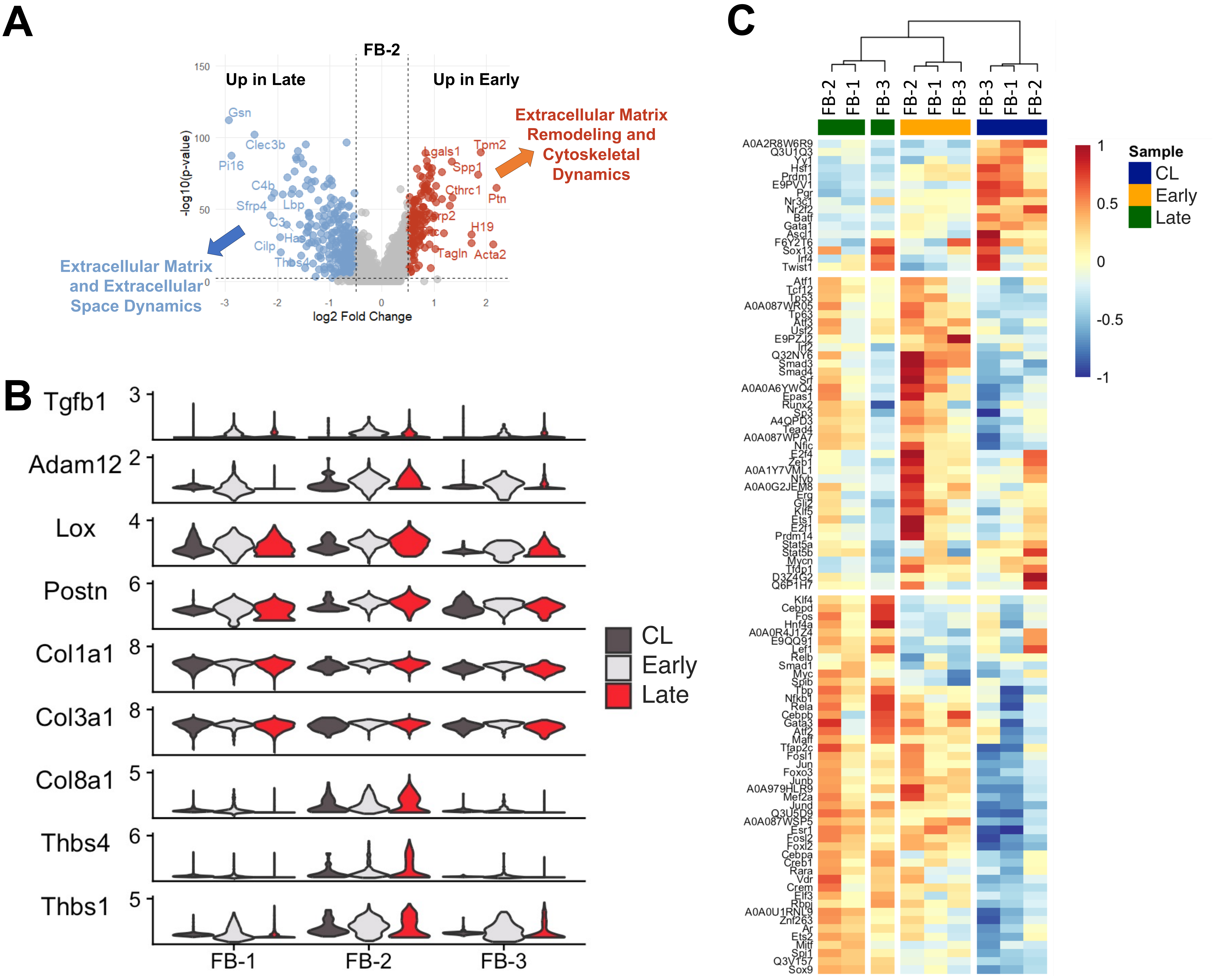

Figure S6. Differentiation of venous fibroblasts after creation of the mouse arteriovenous fistula (AVF). A) Volcano plot of differentially expressed genes in myofibroblasts (FB-2) between early and late AVFs. **B)** Expression changes of selected matrisome genes in the three fibroblast phenotypes during remodeling of the mouse AVF. **C)** Clustered heatmap of the top 100 transcription factors (TFs) per fibroblast phenotype and experimental group. The activity of TFs was predicted based on the expression of target genes. The highest activity of TGF-β signaling factors was detected in early myofibroblasts, while NF-kB factors were most active in late fibroblasts.

Figure S7

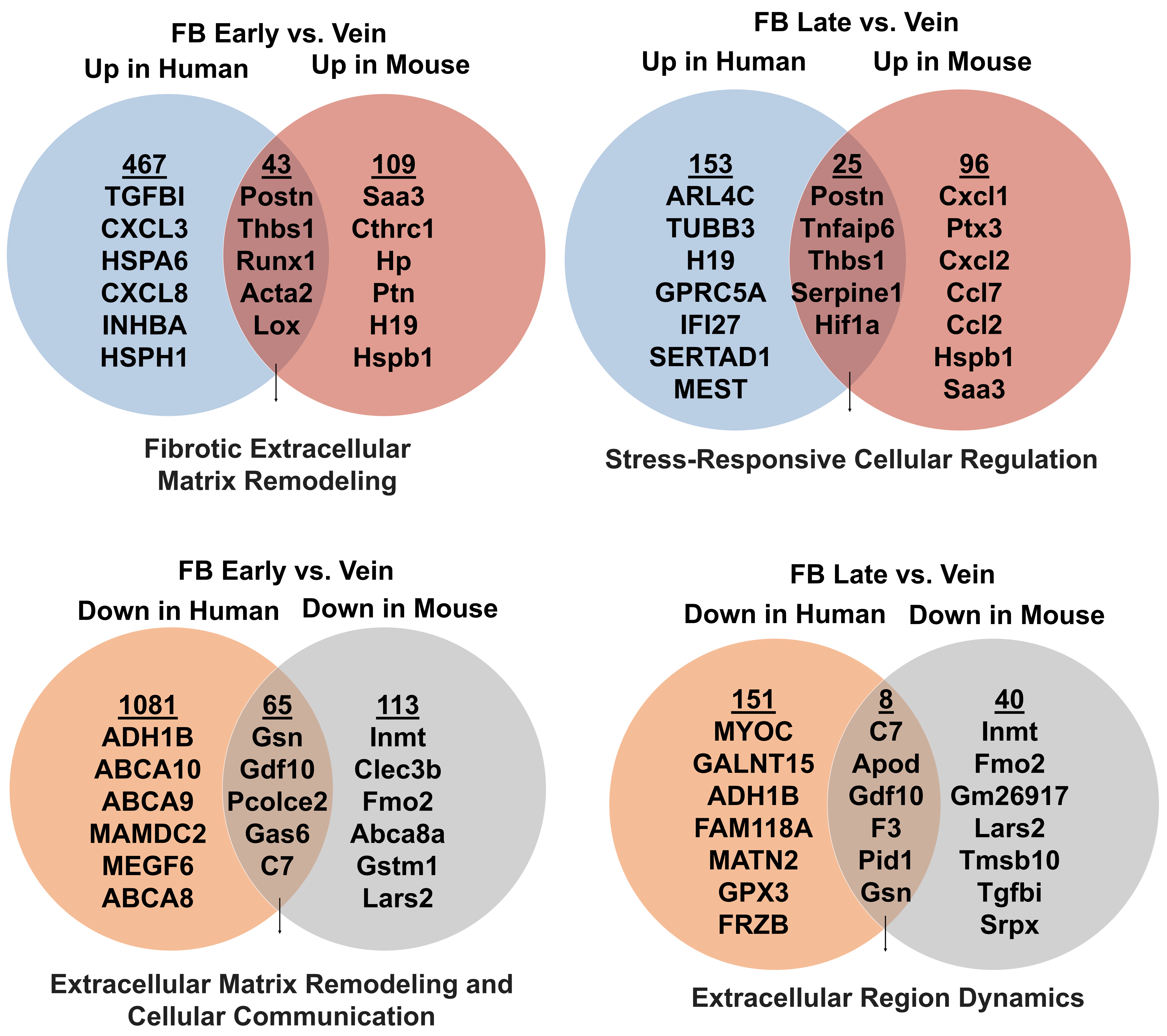

Figure S7. Common differentially expressed genes (DEG) in fibroblasts (FB) from mouse and human arteriovenous fistulas (AVF). The top Venn diagrams show upregulated genes in murine early and late AVFs compared with the contralateral veins, and their intersect with upregulated genes in early and late human fistulas compared with pre-access veins. Similarly, the diagrams at the bottom show the cross-species comparative analyses for the downregulated genes.

Figure S8

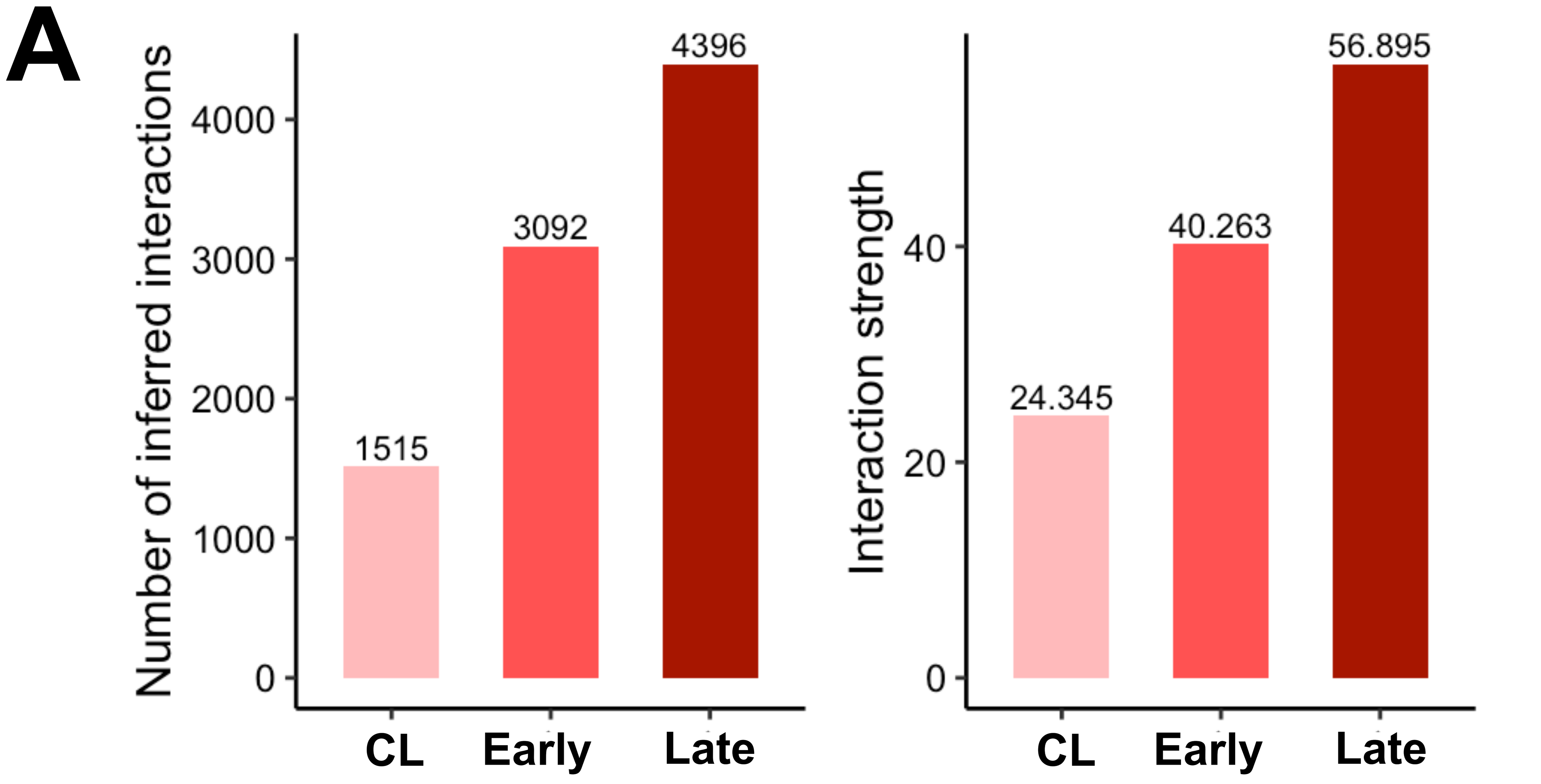

Figure S8. Comparative cell-to-cell communication analyses in the mouse arteriovenous fistula (AVF) using CellChat. A) Number and strength of predicted cellular interactions in the three experimental groups. **B)** Dot plot of outgoing ligands from individual cell populations in contralateral veins (CL) and outflow veins from early and late AVFs. The size of the dot is proportional to the contribution score, with a larger size indicating higher enrichment of the interaction in the cell population.

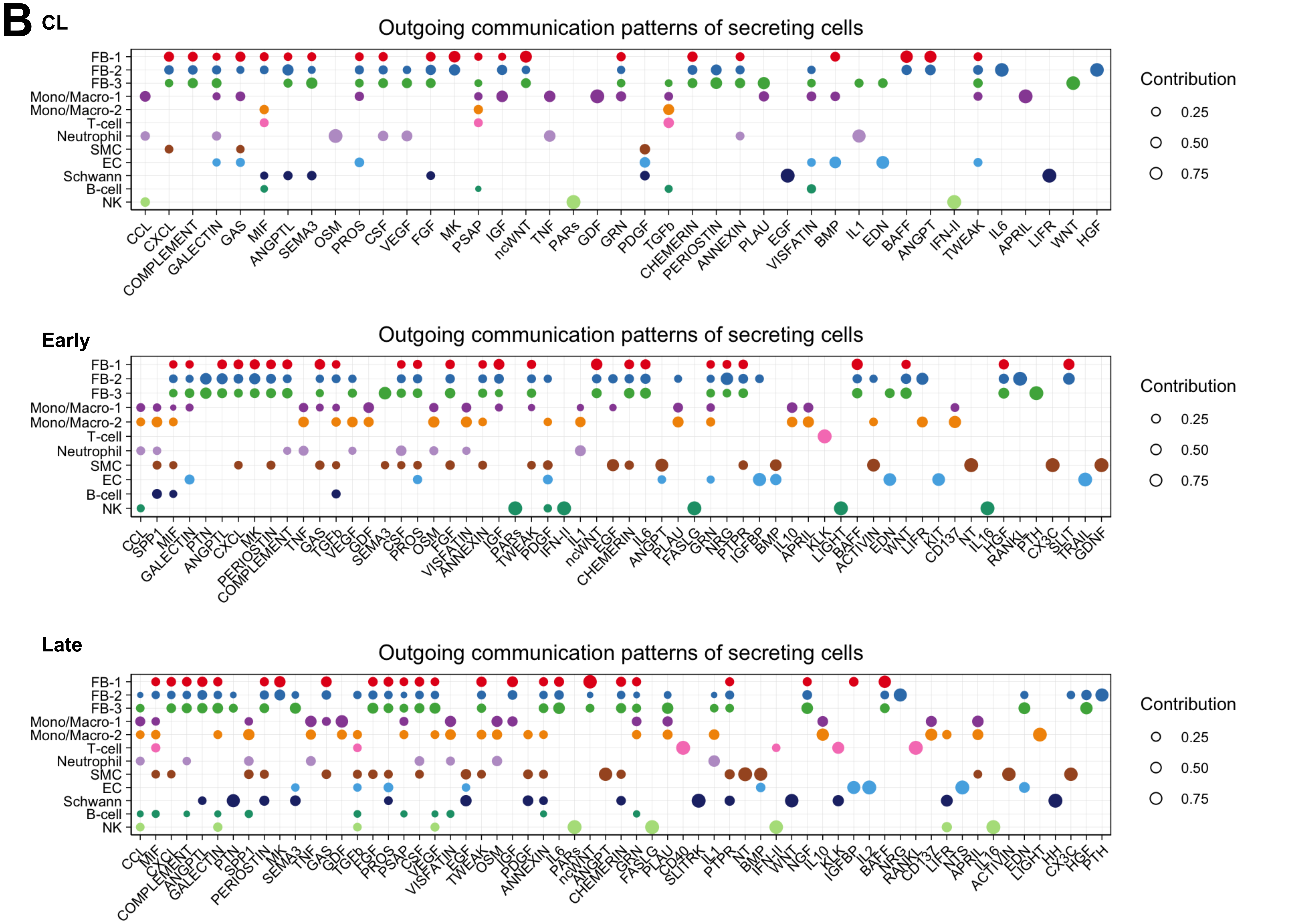

Figure S9

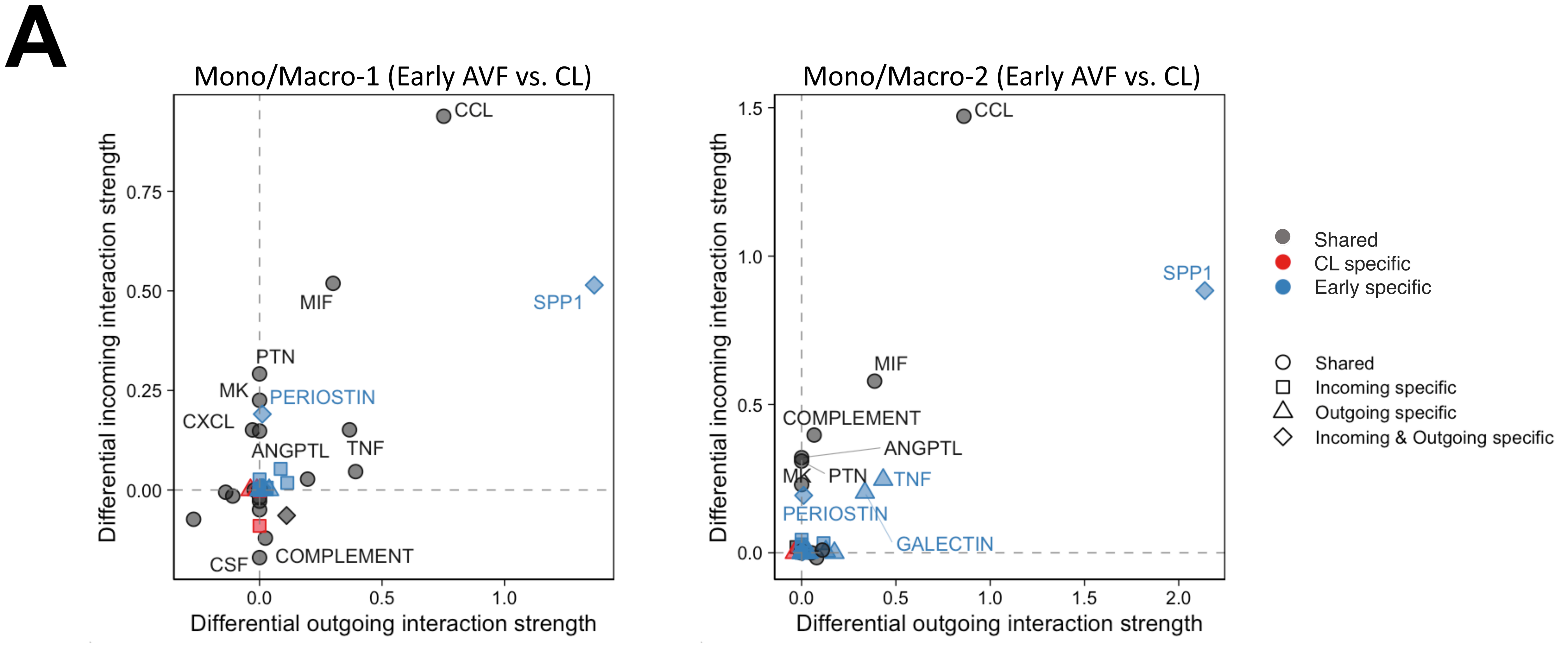

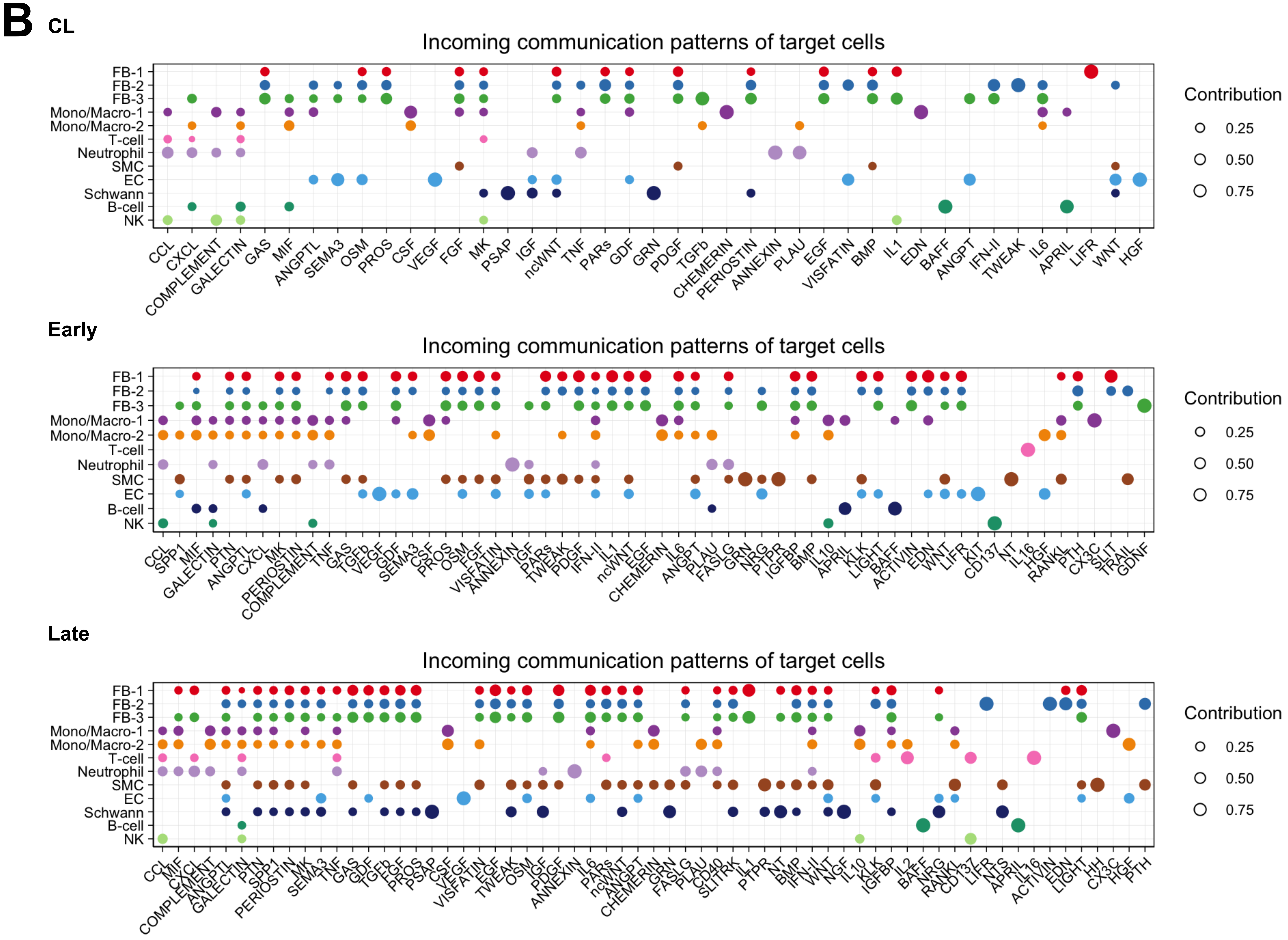

Figure S9. Cell-to-cell communication analyses in the mouse arteriovenous fistula (AVF). A) Differential strength of incoming and outgoing interactions in mono/macros from early AVFs compared with contralateral veins (CL). The top right quadrant includes interactions that are enriched in early AVFs. **B)** Dot plot of incoming ligands to cell populations of contralateral veins (CL) and outflow veins from early and late AVFs.
